# Total resuscitative endovascular balloon occlusion of the aorta causes inflammatory activation and organ damage within 30 minutes of occlusion in normovolemic pigs

**DOI:** 10.1186/s12893-020-00700-3

**Published:** 2020-03-02

**Authors:** Mitra Sadeghi, Emanuel M. Dogan, Christina Karlsson, Kjell Jansson, Jenny Seilitz, Per Skoog, Tal M. Hörer, Kristofer F. Nilsson

**Affiliations:** 1 Department of Cardiothoracic and Vascular Surgery, Faculty of Medicine and Health, Örebro University, SE-70185 Örebro, Sweden; 2 Department of Anesthesiology and Intensive Care, Faculty of Medicine and Health, Örebro University, Örebro, Sweden; 3 School of Health Sciences, Örebro University, Örebro, Sweden; 4 Department of Surgery, Faculty of Medicine and Health, Örebro University, Örebro, Sweden; 5 Department of Vascular Surgery and Institute of Medicine, Department of Molecular and Clinical Medicine, Sahlgrenska University Hospital and Academy, Gothenburg, Sweden

**Keywords:** REBOA, Occlusion time, Ischemia reperfusion injury, Organ damage

## Abstract

**Background:**

Resuscitative endovascular balloon occlusion of the aorta (REBOA) causes physiological, metabolic, end-organ and inflammatory changes that need to be addressed for better management of severely injured patients. The aim of this study was to investigate occlusion time-dependent metabolic, end-organ and inflammatory effects of total REBOA in Zone I in a normovolemic animal model.

**Methods:**

Twenty-four pigs (25-35 kg) were randomized to total occlusion REBOA in Zone I for either 15, 30, 60 min (REBOA15, REBOA30, and REBOA60, respectively) or to a control group, followed by 3-h reperfusion. Hemodynamic variables, metabolic and inflammatory response, intraperitoneal and intrahepatic microdialysis, and plasma markers of end-organ injuries were measured during intervention and reperfusion. Intestinal histopathology was performed.

**Results:**

Mean arterial pressure and cardiac output increased significantly in all REBOA groups during occlusion and blood flow in the superior mesenteric artery and urinary production subsided during intervention. Metabolic acidosis with increased intraperitoneal and intrahepatic concentrations of lactate and glycerol was most pronounced in REBOA30 and REBOA60 during reperfusion and did not normalize at the end of reperfusion in REBOA60. Inflammatory response showed a significant and persistent increase of pro- and anti-inflammatory cytokines during reperfusion in REBOA30 and was most pronounced in REBOA60. Plasma concentrations of liver, kidney, pancreatic and skeletal muscle enzymes were significantly increased at the end of reperfusion in REBOA30 and REBOA60. Significant intestinal mucosal damage was present in REBOA30 and REBOA60.

**Conclusion:**

Total REBOA caused severe systemic and intra-abdominal metabolic disturbances, organ damage and inflammatory activation already at 30 min of occlusion.

## Background

Resuscitative endovascular balloon occlusion of the aorta (REBOA) is an aortic occlusive method using endovascular principles for temporary bleeding control and for increasing perfusion pressure in coronary and cerebral arteries in trauma patients until definitive surgical repair is performed [1–9]. The inflammatory response to aortic occlusion is believed to cause multiple organ failure and late mortality secondary to ischemia reperfusion injuries (IRI) [1, 10–13]. Ischemia causes anaerobic metabolism and impaired cellular membrane function leading to cellular swelling. Reperfusion upregulates the production of oxygen free radicals causing complement system activation and synthesis of endothelial adhesion molecules. IRI is a result of an inflammatory cascade mediated by hormones, proteins and mediators such as interleukine-1 (IL-1), IL-6 and TNF-alfa [10, 11, 14]. It has been suggested that the tissues suffering most from IRI are the kidneys, gastro-intestinal tract and the lower extremities, with virtually all organs being susceptible to the consequences of IRI in aortic occlusion [1, 10]. However, multi-trauma patients are exposed to varying degrees of hypoperfusion and reperfusion, thus complicating the evaluation of the inflammatory response that might be expected from aortic occlusion per se [11, 15, 16]. To date, multiple clinical studies and translational hemorrhagic models have been conducted describing the effects of REBOA in a state of hemorrhagic shock, but few studies have been performed that capture the effects of REBOA in a non-shock condition and give a detailed description of the changes occurring at as early as 15 min and up to 60 min of occlusion [7, 8, 17–21].

Ischemia reperfusion injuries probably occur as a gradual response to aortic occlusion. Even shorter occlusion times than those suggested in previous studies could induce alterations such as endothelial dysfunction, inflammatory activation and organ injury [15, 16, 22]. We hypothesized that significant metabolic and inflammatory response and tissue damage begin already after 15–30 min of total thoracic (Zone I) REBOA. The aim of this study was to investigate potential gradual changes in response to occlusion time on systemic and intra-abdominal metabolic, inflammatory and organ damage variables with Zone I total REBOA in a non-shock porcine model.

## Methods

### Animals

The study was approved by the Linköping Animal Ethics Committee (Linköping, Sweden, ID 105/10) and conducted in accordance with the guidelines of the European Union for the protection of animals used for scientific purposes [23]. The study was undertaken during November 2014 at a research laboratory at the Örebro University Hospital, Örebro, Sweden. Twenty-five pigs (a crossbreed between Swedish country breed, Hampshire and Yorkshire; 3–4 months old; weight range 25–35 kg) of both sexes were used for the experiment. Consent to participate was obtained from the farmer. The animals had free access to food and water before experimentation.

The dataset used in this article are available from the corresponding author on request.

### Anesthesia

Premedication, general anesthesia, ventilation, antibiotic prophylaxis and euthanization at the end of the experiment were recently described [21]. However, one difference was that intravenous bolus doses of pethidine (25–50 mg h^− 1^) were used in this study instead of fentanyl infusion. In brief, the animals were premedicated with azaperone (intramuscular injection) at the farm and transported to the laboratory. General anesthesia was induced by an intramuscular injection of a mixture of azaperone, tiletamine and zolazepam, and maintained by continuous intravenous infusion of propofol and intermittent intravenous boluses of pethidine (25–50 mg h^− 1^). Cefuroxime and atropine were also administered intravenously and intramuscularly, respectively. After endotracheal intubation, the animals were mechanically normoventilated with a positive end-expiratory pressure of 5 cmH_2_O. Fluid loss was substituted with continuous intravenous infusions of Ringer’s acetate and glucose solutions. Body temperature was kept between 37 and 39 °C using forced-air warming blankets. At the end of the experiments, the animals were euthanized with fast intravenous injections of propofol (200 mg, as an overdose of anesthetics) and potassium chloride (40 mmol, to induce cardiac arrest), and ECG confirmed asystole.

### Surgical preparation

The basic surgical preparation and measurements have been recently described in detail [21]. In short, a pulmonary arterial catheter was used for measurements of cardiac output (CO), pulmonary wedge pressure and central venous pressure, and for sampling of mixed venous blood. The common carotid artery catheter was used to measure systemic blood pressure and heart rate (HR), and for arterial blood sampling above the occlusion balloon. The right common femoral artery (CFA) was surgically exposed and used for REBOA introduction and arterial blood sampling below the balloon. The left CFA and superior mesenteric artery (SMA) were dissected, and transonic flow measurement probes were placed around them to measure distal blood flow. A catheter was placed in the superior mesentery vein for measurement of mesenteric venous pressure and for blood sampling. A urinary catheter was placed in the urinary bladder for measuring urinary output and taking urine samples. Microdialysis (mDialysis M62, Sweden) catheters (M70 catheter) were placed intraperitoneally, in the left lower side of the abdomen, and intrahepatically for analysis of extracellular concentrations of glucose, glycerol, lactate and pyruvate.

### Experimental protocol

Before intervention, the animals were each randomized, using envelopes, into one of four groups: 15 min REBOA (REBOA15), 30 min REBOA (REBOA30), 60 min REBOA (REBOA60) in the thoracic descending aorta (Zone I) and a control group (no REBOA, C). The REBOA catheter (Rescue Balloon, 7 Fr, Tokai Medical Products Inc., Kasugai, Japan) was retrogradely advanced with fluoroscopic guidance. After the occlusion, the balloon was slowly deflated over a period of 2 min but not removed. The animals were observed for 3 h after the intervention and hemodynamic variables, blood gases, serum for inflammatory markers, and end organ metabolic enzymes via arterial blood samples were examined. During reperfusion, the animals received only crystalloid fluids, and no vasopressors or inotropic drugs were used. So as not to interfere with the physiological response, the same amount of fluid was given to all groups.

Temperature, urinary output and blood gas analysis from the carotid artery and mesenteric vein were monitored. Intraperitoneal and intrahepatic metabolism was measured using microdialysis catheters, including analysis of glucose, lactate, pyruvate and lactate-pyruvate ratio, and also glycerol as a marker of cellular damage [21]. The inflammatory response was measured by collecting arterial carotid blood in test tubes, allowing coagulation for 30 min followed by centrifugation to achieve serum. The serum was then stored at − 80 °C until analysis. Cytokine detection in serum was performed as previously described [21]. Tissue samples from the small bowel (2 cm × 2 cm) were taken, placed in formalin solution and stained with hematoxylin and eosin stain [21]. The pathologist was blind to the randomization of the animals, and analysis of the severity of the histological changes was assessed using a six-grade system with grade 0 representing normal tissue and grade 5 necrosis [24, 25].

### Statistical analysis

IBM SPSS version 23 was used for statistical analysis. Data are presented as mean and 95% confidence interval. For analysis of variance, a linear mixed model analysis was performed where the repeated factor was time and the other factor was group. If values were significant, post-hoc multiple comparisons were performed using Bonferroni correction. Normal distribution was investigated with the Shapiro-Wilk test. For non-normal distribution, log10 transformation was used, mainly for inflammatory markers. Statistical significance was considered at *p* < 0.05.

## Results

One animal in the REBOA60 group died after deflation of REBOA, probably due to hemodynamic instability, and was replaced. Twenty-four animals completed the experiment with six animals in each group. No significant differences were observed at baseline between the groups.

### Hemodynamic and respiratory variables

**Fig. 1:**
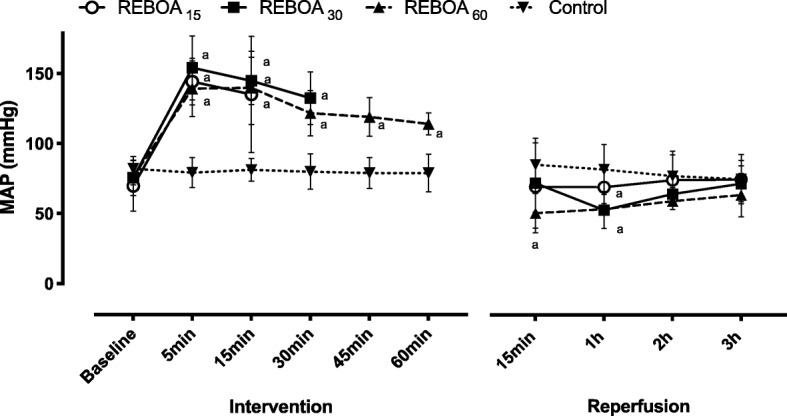
Mean systemic arterial blood pressure (MAP) in anesthetized pigs subjected to Zone I resuscitative endovascular balloon occlusion of the aorta for 15 min (REBOA15), 30 min (REBOA30), 60 min (REBOA60) and control conditions (no occlusion) and reperfusion for 3 h (*n* = 6 per group). Data are means and 95% confidence intervals. a Statistically significant difference compared to controls

Aortic occlusion in all REBOA groups induced immediate systemic hypertension, tachycardia and increased CO (Table 1, Fig. 1). These effects gradually subsided throughout the occlusion period in all REBOA groups, with these changes being correspondingly more pronounced in REBOA60 than in REBOA15 at the end of the occlusion period (Table 1, Fig. 1). During aortic occlusion, the blood flows in SMA and CFA ceased completely in all REBOA groups (Table 1). At reperfusion, the systemic arterial blood pressure decreased in all REBOA groups, however to a lower level in REBOA60 compared to REBOA15 and REBOA30 (Fig. 1, Table 1). Heart rate and CO returned towards control group values during the reperfusion period, but were non-significantly higher and lower, respectively, in REBOA60 compared to REBOA15 and REBOA30 (Table 1). Blood flow in SMA and CFA increased towards baseline values during the reperfusion period, however less in REBOA60 (Table 1). The central hemodynamic and blood flow variables were maintained in the control group (Fig. 1, Table 1). Minute ventilation and fraction of inspired oxygen (FiO_2_) were similar in all groups and constant over time (data not shown). The PaO_2_/FiO_2_ ratio was unchanged and similar in all groups (Table 1). In all REBOA groups, end-tidal carbon dioxide (ETCO_2_) was significantly reduced during aortic occlusion but normalized during reperfusion (Table 1).

**Table 1:** Hemodynamic and respiratory variables in anesthetized pigs subjected to Zone I resuscitative endovascular balloon occlusion of the aorta for 15 (REBOA15), 30 (REBOA30), 60 min (REBOA60) and control conditions (no occlusion) and reperfusion for 3 h (*n* = 6 per group)

Variables	Baseline	End of intervention	15 min reperfusion	1 h reperfusion	2 h reperfusion	3 h reperfusion
Heart rate (beats min^−1^)						
Control	106 (91–121)	102 (85–120)^bcd^	103 (86–120)^bcd^	102 (83–122)^b^	99 (80–117)	96 (78–114)
REBOA15	120 (78–162)	209 (185–232)^a^	169 (156–181)^a^	152 (119–185)^a^	131 (93–170)	128 (98–159)
REBOA30	110 (90–129)	213 (196–230)^a^	171 (145–198)^a^	139 (109–170)	126 (97–155)	129 (110–149)
REBOA60	122 (65–179)	190 (168–212)^a^	157 (125–189)^a^	133 (100–166)	136 (101–172)	140 (100–181)
SBP (mmHg)						
Control	102 (97–106)	96 (84–107)^bcd^	98 (89–106)	95 (83–107)	91 (80–102)	88 (76–99)
REBOA15	94 (79–110)	194 (136–252)^ad^	106 (91–121)	92 (77–106)	95 (82–109)	94 (82–105)
REBOA30	97 (80–114)	170 (140–200)^a^	108 (85–131)^d^	84 (75–94)	86 (77–95)	89 (69–108)
REBOA60	96 (91–101)	146 (128–164)^ab^	80 (65–95)^c^	81 (75–88)	85 (81–89)	91 (78–104)
CO (l min^−1^)						
Control	4.9 (3.7–6.1)	4.6 (3.6–5.6)^bc^	4.5 (3.5–5.6)	4.4 (3.4–5.3)	4.3 (3.5–5.1)	4.3 (3.4–5.1)
REBOA15	4.9 (3.8–6.1)	6.1 (5.4–6.8)^ad^	5.8 (4.4–7.1)^d^	4.8 (3.7–5.9)	4.7 (3.5–5.8)	4.5 (3.6–5.4)
REBOA30	4.7 (3.7–5.6)	6.1 (5.5–6.8)^ad^	5.4 (4.7–6.1)^d^	4.5 (3.6–5.4)	4.1 (3.4–4.8)	4.3 (3.5–5.0)
REBOA60	4.1 (3.2–4.9)	4.2 (3.8–4.7)^bc^	3.8 (2.7–4.9)^bc^	3.7 (3.0–4.3)	3.4 (2.9–4.0)	3.3 (2.6–3.9)
SMA blood flow (ml min^−1^)						
Control	797 (605–990)	771 (614–928)^bcd^	755 (617–893)	734 (581–886)	709 (530–887)	704 (515–894)
REBOA15	747 (479–1015)	35 (18–52)^a^	910 (654–1165)	873 (565–1180)	830 (439–1220)	784 (451–1117)
REBOA30	731 (434–1027)	17 (9–24)^a^	915 (691–1138)	772 (528–1016)	733 (520–946)	687 (467–907)
REBOA60	642 (458–826)	21 (10–33) ^a^	617 (305–929)	723 (479–967)	633 (487–780)	572 (408–736)
Femoral blood flow (ml min^−1^)						
Control	266 (147–386)	228 (116–341)^bcd^	225 (105–345)	212 (110–313)	207 (116–297)	199 (111–286)
REBOA15	287 (203–371)	14 (1–26)^a^	242 (97–387)	234 (110–358)	204 (94–315)	199 (95–303)
REBOA30	347 (181–513)	19 (2–35)^a^	328 (172–483)	280 (175–384)	256 (180–333)	243 (168–317)
REBOA60	289 (178–400)	15 (−4–34)^a^	221 (55–386)	176 (79–272)	149 (39–259)	169 (47–291)
ETCO_2_ (%)						
Control	5.0 (4.7–5.3)	4.7 (4.2–5.1)^bcd^	4.6 (4.3–4.9) ^c^	4.6 (4.3–4.9)	4.6 (4.1–5.0)	4.4 (4.0–4.7)
REBOA15	4.9 (4.6–5.1)	3.5 (3.2–3.8)^a^	5.0 (4.7–5.2)^b^	4.9 (4.6–5.3)	4.9 (4.8–5.0)	4.6 (4.2–4.9)
REBOA30	4.9 (4.5–5.3)	3.4 (3.0–3.8)^a^	5.8 (5.2–6.5)^abd^	5.1 (4.5–5.7)	4.7 (4.4–5.0)	4.7 (4.3–5.1)
REBOA60	4.7 (4.5–4.9)	2.9 (2.3–3.6)^a^	4.8 (3.8–5.7)^c^	4.6 (3.8–5.4)	4.6 (4.0–5.1)	4.5 (3.4–5.5)
FiO_2_ (%)						
Control	26 (23–29)	26 (23–29)	28 (25–30)	28 (25–30)	28 (25–30)	28 (26–30)
REBOA15	25 (23–27)	26 (24–28)	27 (23–31)	27 (24–31)	28 (23–32)	27 (23–31)
REBOA30	26 (22–29)	26 (23–29)	26 (22–29)	26 (22–29)	28 (22–33)	28 (23–34)
REBOA60	29 (23–35)	29 (21–36)	29 (22–36)	30 (24–36)	29 (25–33)	29 (24–33)
PaO_2_/FiO_2_						
Control	64 (57–71)	61 (53–69)^d^	64 (58–69)	64 (58–71)	67 (63–72)	66 (63–70)
REBOA15	64 (56–72)	71 (63–79)	64 (54–75)	66 (60–71)	65 (54–76)	68 (57–79)
REBOA30	66 (61–71)	73 (66–79)	57 (49–65)	60 (55–65)	68 (62–75)	67 (61–73)
REBOA60	71 (60–81)	73 (64–83)^a^	64 (53–75)	69 (59–80)	69 (62–76)	67 (58–76)
Mesenteric oxygen uptake VO_2_ (ml O_2_ min^−1^)						
Control	41 (26–56)	36 (18–53)^bcd^		37 (27–46)		35 (23–47)
REBOA15	36 (27–46)	3.6 (1.0–6.3)^a^		38 (25–52)		29 (10–48)
REBOA30	29 (13–44)	1.7 (1.0–2.4)^a^		35 (2.5–67)		31 (5.7–56)
REBOA60	28 (17–40)	2.0 (1.0–2.9)^a^		24 (10–38)		23 (17–29)

Data are means (95% confidence interval). *SBP* Systolic arterial blood pressure, *CO* Cardiac output, *SMA* Superior mesenteric artery, *ETCO*_*2*_ End-tidal carbon dioxide, *FiO*_*2*_ Fraction of inspired oxygen, *PaO*_*2*_ arterial partial pressure of oxygen, *VO*_*2*_ Oxygen uptake

^a^ Statistically significant difference compared to controls

^b^ Statistically significant difference compared to REBOA 15 min

^c^ Statistically significant difference compared to REBOA 30 min

^d^ Statistically significant difference compared to REBOA 60 min

### Systemic acid-base status and potassium

**Fig. 2:**
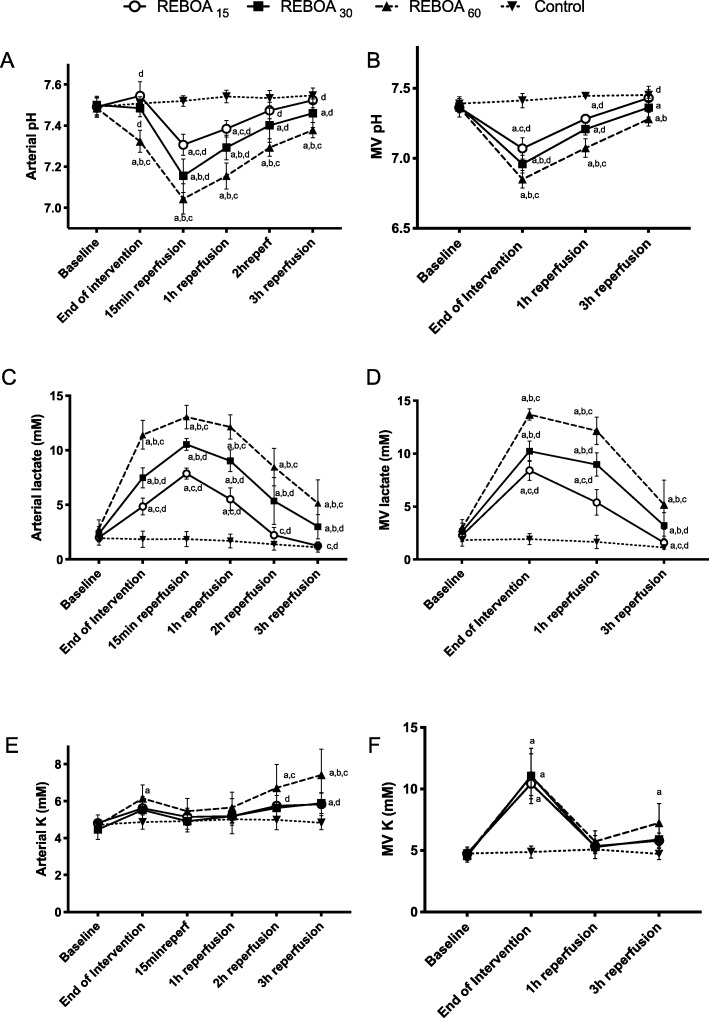
Systemic and mesenteric venous (MV) pH (panel **a**, **b**), lactate concentration (panel **c**, **d**) and potassium concentration (panel **e**, **f**) in anesthetized pigs subjected to zone I resuscitative endovascular balloon occlusion of the aorta for 15 (REBOA15), 30 (REBOA30), 60 min (REBOA60) and control conditions (no occlusion) and reperfusion for 3 h (*n* = 6 per group). Data are means and 95% confidence intervals. **a** Statistically significant difference compared to control, **b** Statistically significant difference compared to REBOA 15 min, **c** Statistically significant difference compared to REBOA 30 min, **d** Statistically significant difference compared to REBOA 60 min

At the end of aortic occlusion, arterial pH was unchanged in REBOA15 and REBOA30 compared to controls but was significantly lower in REBOA60 (Fig. 2). Arterial lactate concentrations were significantly different between all REBOA groups, with the most pronounced increase in REBOA60 and arterial potassium concentrations were significantly higher in REBOA60 compared to control at the end of aortic occlusion (Fig. 2). During early reperfusion, arterial pH decreased and arterial lactate concentrations increased in all REBOA groups, and the magnitude of these changes was dependent on duration of aortic occlusion (Fig. 2). During the reperfusion period, arterial pH and lactate concentrations slowly returned to baseline values, however they were only normalized in REBOA15 (Fig. 2). Arterial potassium concentrations increased steadily throughout the reperfusion period in REBOA60, and were significantly higher at the end of reperfusion compared to the other REBOA groups (Fig. 2).

### Intra-abdominal metabolism

Mesenteric oxygen uptake was significantly reduced during occlusion in all groups but normalized during reperfusion (Table 1). Mesenteric venous pH was reduced, and mesenteric venous lactate and potassium concentrations were increased during occlusion in all REBOA groups; these changes were most pronounced in REBOA60. These returned towards normal during reperfusion in all groups but did not completely recover in REBOA30 and REBOA60 (Fig. 2).

**Fig. 3:**
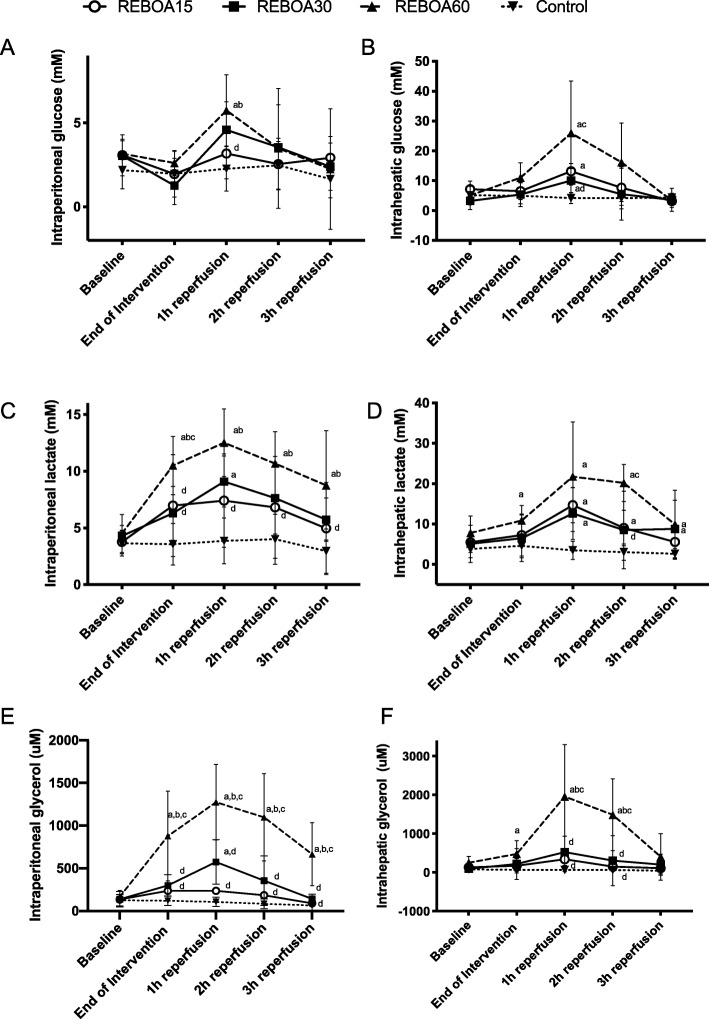
Intraperitoneal and intrahepatic concentrations of glucose (panel **a**, **b**), lactate (panel **c**, **d**) and glycerol (panel **e**, **f**) in anesthetized pigs subjected to Zone I resuscitative endovascular balloon occlusion of the aorta for 15 min (REBOA15), 30 min (REBOA30), 60 min (REBOA60) and control conditions (no occlusion) and reperfusion for 3 h (*n* = 6 per group). Data are means and 95% confidence intervals. a Statistically significant difference compared to controls, b Statistically significant difference compared to REBOA 15 min, c Statistically significant difference compared to REBOA 30 min, d Statistically significant difference compared to REBOA 60 min

Intraperitoneal and intrahepatic concentrations of lactate increased during aortic occlusion, peaked at 1 h of reperfusion and only returned to baseline values at the end of the experiment in REBOA15 (Fig. 3). The concentration of lactate was mostly increased in REBOA60 and was higher intrahepatically than intraperitoneally. The intraperitoneal lactate-pyruvate ratio was increased during aortic occlusion in REBOA30 (27 [− 1.5–56) and REBOA60 (26 [18–34]) compared to controls (16 [9–23]), but returned towards normal. The intrahepatic lactate-pyruvate ratio as mentioned increased more than intraperitoneal in REBOA30 (46 [− 56–147]) and REBOA60 (116 [− 115–346]) compared to controls (31 [10–52]) The intraperitoneal and intrahepatic concentrations of glucose were unchanged during aortic occlusion in all REBOA groups and increased during reperfusion, reaching the highest levels at 1 h of reperfusion and being most pronounced in REBOA60 in the intrahepatic compartment. At the end of reperfusion, the concentrations returned to baseline values in all REBOA groups (Fig. 3).

### Visceral organs

At the end of reperfusion, plasma creatinine, aspartate aminotransferase and lipase concentrations were significantly increased in REBOA30 and REBOA60, but not in REBOA15, compared to controls (Table 2). The plasma alanine aminotransferase and creatine kinase concentrations increased most, and significantly in REBOA60 during reperfusion compared to the other groups (Table 2). Urinary output decreased to zero in all REBOA groups during occlusion (Table 1). The intraperitoneal concentrations of glycerol, a marker of cell membrane damage, started to increase during occlusion in all REBOA groups and increased significantly throughout reperfusion, being most pronounced in REBOA60 (Fig. 3). Intrahepatic concentrations of glycerol were only increased during early reperfusion in REBOA60 (Fig. 3).

**Table 2:** End-organ damage markers in anesthetized pigs subjected to Zone I resuscitative endovascular balloon occlusion of the aorta for 15 min (REBOA15), 30 min (REBOA30), 60 min (REBOA60) and control conditions (no occlusion) and reperfusion for 3 h (*n* = 6 per group)

Variables	Baseline	1 h reperfusion	3 h reperfusion
P-ALT (μkat l^− 1^)			
Control	1.4 (1.1–1.7)	1.4 (1.1–1.7)	1.3 (1.1–1.6)
REBOA15	1.3 (1.1–1.5)	1.2 (1.0–1.4)	1.2 (1.0–1.5)^d^
REBOA30	1.3 (1.1–1.6)	1.3 (1.0–1.5)	1.5 (1.2–1.7)
REBOA60	1.5 (1.2–1.8)	1.4 (1.0–1.7)	1.7 (1.3–2.1)^b^
P-AST (μkat l^−1^)			
Control	1.2 (0.9–1.5)	1.2 (0.7–1.6)^d^	1.1 (0.6–1.6)^cd^
REBOA15	1.1 (0.9–1.3)	1.2 (1.0–1.3)^d^	1.3 (1.0–1.5) ^cd^
REBOA30	1.3 (0.8–1.8)	1.8 (1.2–2.3)	2.5 (2.1–3.0)^abd^
REBOA60	1.3 (1.0–1.6)	2.4 (1.9–3.0)^ab^	4.3 (2.4–6.3)^abc^
P-CK (μkat l^−1^)			
Control	15 (12–18)	14 (12–17)^d^	13 (11–15)^d^
REBOA15	18 (11–24)	23 (12–34)	21 (10–31)^d^
REBOA30	17 (13–21)	22 (16–28)	23 (16–30)^d^
REBOA60	22 (15–29)	29 (20–39)^a^	37 (26–47)^abc^
P-Creatinine (μmol l^−1^)			
Control	86 (58–115)	90 (63–116)^d^	94 (74–114)^cd^
REBOA15	75 (66–83)	91 (78–104)^d^	93 (82–104)^cd^
REBOA30	82 (76–88)	116 (102–129)	123 (109–137)^ab^
REBOA60	91 (72–110)	136 (110–161)^b^	151 (126–175)^ab^
P-Lipase (μkat l^−1^)			
Control	0.4 (0.2–0.5)	0.2 (0.2–0.3)^d^	0.2 (0.2–0.3)^cd^
REBOA15	0.4 (0.2–0.6)	0.5 (0.2–0.7)	0.4 (0.1–0.7)^d^
REBOA30	0.5 (0.3–0.7)	0.8 (0.4–1.3)	1.3 (0.6–2.0)^ad^
REBOA60	0.4 (0.2–0.7)	1.5 (0.6–2.3)^a^	3.0 (1.2–4.9)^abc^
Urinary output (ml h^−1^)			
Control	33 (8–58)	59 (−11–128)	60 (19–101)
REBOA15	33 (10–55)	62 (−36–159)	93 (32–153)
REBOA30	27 (16–38)	21 (0–43)	46 (10–82)
REBOA60	33 (18–49)	17 (−1–35)	65 (−8–138)

Data are means (95% confidence interval). *ALT* Alanine aminotransferase, *AST* Aspartate aminotransferase, *CK* Creatine kinase

^a^ Statistically significant difference compared to controls

^b^ Statistically significant difference compared to REBOA 15 min

^c^ Statistically significant difference compared to REBOA 30 min

^d^ Statistically significant difference compared to REBOA 60 min

### Cytokine response

**Fig. 4:**
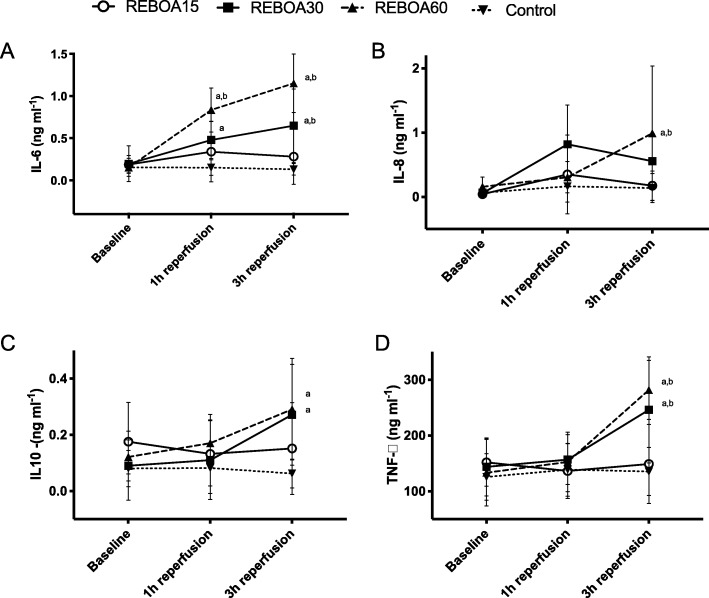
Plasma concentrations of interleukin-6 (IL-6, panel **a**), interleukin-8 (IL-8, panel **b**), interleukin-10 (IL-10, panel **c**) and tumor necrosis factor alpha (TNF-α, panel **d**) in anesthetized pigs subjected to Zone I resuscitative endovascular balloon occlusion of the aorta (I) for 15 min (REBOA15), 30 min (REBOA30), 60 min (REBOA60) and control conditions (no occlusion) and reperfusion for 3 h (*n* = 6 per group). Data are means and 95% confidence intervals. **a** Statistically significant difference compared to controls. **b** Statistically significant difference compared to REBOA 15 min. **c** Statistically significant difference compared to REBOA 30 min. **d** Statistically significant difference compared to REBOA 60 min

Pro-inflammatory interleukins (IL-6, IL-8, IL-1β and TNF-alfa) and the anti-inflammatory interleukin (IL-10) increased at 1 h of reperfusion in REBOA15 but normalized at the end of reperfusion (Fig. 4 and data not shown). In REBOA30 and REBOA60, there was a continuous increase of the interleukin concentrations throughout reperfusion (Fig. 4).

### Histology

**Fig. 5:**
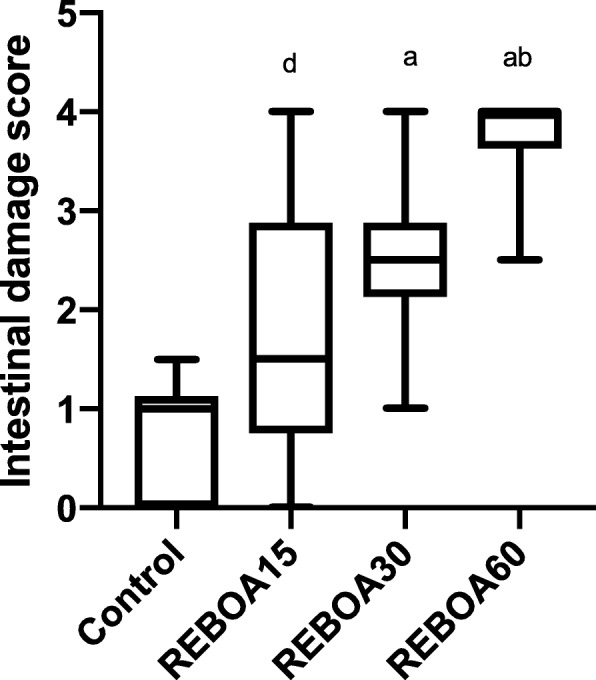
Histological mucosal damage score in anesthetized pigs subjected to Zone I resuscitative endovascular balloon occlusion of the aorta for 15 min (REBOA15), 30 min (REBOA30), 60 min (REBOA60) and control conditions (no occlusion) and reperfusion for 3 h (*n* = 6 per group). Data are means and 95% confidence intervals. **a** Statistically significant difference compared to controls. **b** Statistically significant difference compared to REBOA 15 min. **c** Statistically significant difference compared to REBOA 30 min. **d** Statistically significant difference compared to REBOA 60 min

There was significantly more ischemic damage in REBOA30 and REBOA60 compared to REBOA15 and controls (Fig. 5).

## Discussion

The present study investigated the hemodynamic, metabolic, end-organ and inflammatory effects of REBOA in relation to duration of aortic occlusion in a non-hemorrhagic animal model. The findings regarding the hemodynamic response are consistent with previous studies on thoraco-abdominal aortic clamping [26, 27], but with further and more profound examination of the alterations in metabolic and inflammatory responses in various organs during aortic occlusion and reperfusion. These detailed metabolic findings have not been reported before to the best of our knowledge.

Aortic occlusion caused an immediate increase in systemic arterial blood pressure, which increased by 100% within 5 min of occlusion (not shown in the figures). The observed hemodynamic response is presumably due to different mechanisms of action, such as secretion of catecholamines and renin-angiotensin, blood volume redistribution with absorption of intestinal fluid into the circulation, and venous capacity reduction inducing central hypervolemia. However, systemic blood pressure slowly decreased over time in all REBOA groups starting as early as within 15 min of aortic occlusion. This phenomenon could be explained by reduced return of blood from the splanchnic vasculature, and perhaps over activation of autonomic and hormonal signaling systems, thus suggesting that the desired effect of the aortic balloon occlusion might eventually diminish with longer occlusion times [26, 27]. Ischemia and reperfusion cause reactive hyperemia mediated by the inflammatory response leading to pooling of the blood in the distal parts of the body whereby systemic hypovolemia arises [1], which was most pronounced in REBOA60. This study found no evidence for respiratory failure with increased pulmonary vascular resistance and edema as previously described, i.e. PaO2/FiO2 ratio was not affected [1, 14].

Reduced organ perfusion leads to cellular anaerobic metabolism, which was evident with significantly increased concentrations of lactate in the mesenteric vein during aortic occlusion, reaching maximum levels in arterial blood during early reperfusion as the ischemic blood returns to the circulation. Arterial and mesenteric venous lactate concentrations increased in all REBOA groups, but mostly in REBOA30 and REBOA60, and did not return to baseline values during the 3 h of reperfusion. Intrahepatic lactate concentration increased two-fold compared with intraperitoneal concentration during early reperfusion, possibly due to the gluconeogenesis process in the liver where lactate is metabolized to produce glucose. Similar results were shown with glycerol concentrations. The anaerobic metabolites peaked during early reperfusion and normalized within 3 h of reperfusion in REBOA15, but not in REBOA30 or REBOA60 probably due to greater ischemic insult. During early reperfusion, the concentrations of intraperitoneal and intrahepatic glucose increased in REBOA30 and REBOA60. The increased glucose could be attributed to sympatho-adrenal discharge leading to glycogenolysis, but possibly also to reduced perfusion of the pancreas diminishing insulin release [1].

The inflammatory response associated with multiple organ failure in trauma patients was studied by Svoboda et al. who suggested a correlation between IL-6 levels and injury severity and the development of multiple organ failure [12]. Another study by Wallinder et al. describing early inflammatory response in patients with ruptured abdominal aortic aneurysm (rAAA) showed significantly higher levels of IL-6 and IL-10 in patients with rAAA compared to non-ruptured AAA [28]. Similar findings have been described in experimental hemorrhagic animal models using supraceliac aortic occlusion for 30 to 90 min, with the increase of IL-6, IL1beta, TNF-alfa and IL10 suggesting a correlation between aortic occlusion time, the magnitude of the inflammatory response, and organ failure [11, 14]. The inflammatory response in this study caused by aortic occlusion and reperfusion was observed at as early as 1 h of reperfusion, with a significant increase of IL-6 in REBOA60 and REBOA30 compared to controls. At 1 h of reperfusion, both pro- and anti-inflammatory cytokines increased in REBOA 30 and 60 as a response to aortic occlusion, which became significant by the end of the experiment. The denominator for all these studies is organ hypoperfusion, which activates the inflammatory cascade and is most profound when using an aortic occlusive device such as REBOA. This has to be taken into consideration during resuscitation after REBOA use.

Ischemia-reperfusion injuries to visceral organs were demonstrated with increases in liver- and pancreatic enzymes, reduced kidney filtration and muscle damage throughout reperfusion, with significantly higher enzyme concentrations in REBOA60 but also seen in REBOA30. Significant histological mucosal ischemic injuries were already present in REBOA30.

IRI also caused hyperkalemia in all groups through acidosis and reduced excretion in the kidneys, and reduced insulin levels due to diminished perfusion of the pancreas [1]. This could be a lethal consequence of the ischemia and reperfusion leading to cardiac arrhythmia and death. Interestingly, potassium reached maximum levels during late reperfusion in all groups, being most pronounced in REBOA60, which is a sign of serious organ injury. These findings suggest that organ injury probably begins earlier than previously explained and even 30 min of occlusion causes ischemic insult [29]. To determine a safe aortic occlusion time is probably impossible due to the multiple insults a severely traumatized patient suffers, but the knowledge that only 30 min of aortic occlusion may initiate severe metabolic derangements, end-organ injury and a significant inflammatory response is important. The use of techniques to limit ischemic insults of aortic occlusion, such as the use of partial REBOA, is therefore warranted when possible [30–33]. Future studies from the team will focus on development of partial occlusion technique.

There are some limitations to our experimental model. The short reperfusion time does not allow investigation of irreversible organ damage. However the trends in the immediate post-occlusion period indicates severe metabolic and inflammatory consequences of REBOA use. Another limitation of this study is that it is a non-hemorrhagic model and excludes the metabolic and inflammatory impact of traumatic injury, however the aim of the study was to investigate the consequences of aortic occlusion per se.

## Conclusion

Total REBOA caused severe systemic and intra-abdominal metabolic disturbances and organ damage as well as inflammatory activation already at 30 min of occlusion in a non-hemorrhagic porcine model. Safe aortic occlusion time needs to be re-evaluated. The use of techniques to limit the ischemic insult by REBOA is demanded, even if a relatively short occlusion time is expected.

## Data Availability

Data is available on a reasonable request to the corresponding author.

## Abbreviations

*CFA*: Common femoral artery
*CO*: Cardiac output
*ETCO*_*2*_: End-tidal carbon dioxide
*FiO*_*2*_: Fraction of inspired oxygen
*HR*: Heart rate
*IL*: Interleukine
*IRI*: Ischemia reperfusion injury
*REBOA*: Resuscitative endovascular balloon occlusion of the aorta
*SMA*: Superior mesenteric artery
